# Perspective on the Assessment of Skeletal Muscle Mass

**DOI:** 10.31662/jmaj.2023-0127

**Published:** 2023-09-29

**Authors:** Hidenori Arai

**Affiliations:** 1 National Center for Geriatrics and Gerontology, Obu, Japan

**Keywords:** skeletal muscle, sarcopenia, DXA, BIA

Muraki summarizes the skeletal muscle mass diagnosis and measurement methods for sarcopenia in the study entitled “Muscle Mass Assessment in Sarcopenia: A Narrative Review” ^(1)^. Previously, measurement of skeletal muscle mass was a standalone requirement for the diagnosis of sarcopenia; moreover, it was a requirement in diagnostic criteria such as European Working Group on Sarcopenia in Older People (EWGSOP), European Society for Clinical Nutrition and Metabolism-Special Interest Group (ESPEN-SIG), International Working Group on Sarcopenia (IWGS), and Asian Working Group for Sarcopenia (AWGS), along with grip strength and walking speed. As Muraki indicates, Computed Tomography (CT) and Magnetic Resonance Imaging (MRI) are highly accurate but difficult to implement in clinical and community settings. Conversely, Dual-energy X-ray Absorptiometry (DXA) and Bioelectrical Impedance Analysis (BIA) are easier to implement but have accuracy concerns. D3-creatine dilution and echocardiography are promising in terms of applicability and accuracy, but there is insufficient evidence regarding cutoff values; however, future development is expected.

Furthermore, when estimating skeletal muscle mass with DXA and BIA, correction is made for height squared, weight, and Body Mass Index (BMI), but there is no consensus on which is the most appropriate correction method, although height squared correction has been commonly used. For example, in the case of obesity, correction for height squared tends to overestimate skeletal muscle mass; thus, correction for body weight or BMI is preferable. Although we have demonstrated the utility of skeletal muscle measurement in the longitudinal cohort and clinical studies ^(2), (3), (4)^, Sarcopenia Definitions and Outcomes Consortium (SDOC) has determined that skeletal muscle measurement is not necessary for the diagnosis of sarcopenia ^(5)^. Currently, the Global Leadership Initiative on Sarcopenia (GLIS) group has been formed to globally discuss the definition and diagnostic criteria for sarcopenia. Liang-Kung Chen, Jean Woo, and Hidenori Arai are participating in this group from Asia, and further discussions are expected.

## Article Information

### Conflicts of Interest

None
